# Neural processing of emotions in traumatized children treated with Eye Movement Desensitization and Reprocessing therapy: a hdEEG study

**DOI:** 10.3389/fpsyg.2015.01662

**Published:** 2015-11-05

**Authors:** Cristina Trentini, Marco Pagani, Piercarlo Fania, Anna Maria Speranza, Giampaolo Nicolais, Alessandra Sibilia, Lucio Inguscio, Anna Rita Verardo, Isabel Fernandez, Massimo Ammaniti

**Affiliations:** ^1^Department of Dynamic and Clinical Psychology, “Sapienza” University of RomeRome, Italy; ^2^Institute of Cognitive Sciences and Technologies, National Research Council (CNR)Rome, Italy; ^3^Positron Emission Tomography Center IRMET SpA, Euromedic Inc.Turin, Italy; ^4^Department of Developmental and Social Psychology, “Sapienza” University of RomeRome, Italy; ^5^EMDR Italy AssociationMilan, Italy; ^6^Clinical Centre, Feel SafeRome, Italy; ^7^“Sapienza” University of RomeRome, Italy; ^8^International Psychoanalytical AssociationLondon, UK

**Keywords:** complex trauma, EMDR, hdEEG, emotion processing, children’s emotional-adaptive functioning

## Abstract

Eye Movement Desensitization and Reprocessing (EMDR) therapy has been proven efficacious in restoring affective regulation in post-traumatic stress disorder (PTSD) patients. However, its effectiveness on emotion processing in children with complex trauma has yet to be explored. High density electroencephalography (hdEEG) was used to investigate the effects of EMDR on brain responses to adults’ emotions on children with histories of early maltreatment. Ten school-aged children were examined before (T0) and within one month after the conclusion of EMDR (T1). hdEEGs were recorded while children passively viewed angry, afraid, happy, and neutral faces. Clinical scales were administered at the same time. Correlation analyses were performed to detect brain regions whose activity was linked to children’s traumatic symptom-related and emotional-adaptive problem scores. In all four conditions, hdEEG showed similar significantly higher activity on the right medial prefrontal and fronto-temporal limbic regions at T0, shifting toward the left medial and superior temporal regions at T1. Moreover, significant correlations were found between clinical scales and the same regions whose activity significantly differed between pre- and post-treatment. These preliminary results demonstrate that, after EMDR, children suffering from complex trauma show increased activity in areas implicated in high-order cognitive processing when passively viewing pictures of emotional expressions. These changes are associated with the decrease of depressive and traumatic symptoms, and with the improvement of emotional-adaptive functioning over time.

## Introduction

Post-traumatic stress disorder (PTSD) describes discrete conditioned behavioral and biological responses to an experience involving actual or threatened death, serious injury, or sexual violence. The exposure must result from one or more of the following scenarios, in which the individual: (a) directly experiences the traumatic event; (b) witnesses the traumatic event in person; (c) learns that the traumatic event has occurred to a close family member or close friend (with the actual or threatened death being either violent or accidental); or (d) experiences first-hand repeated or extreme exposure to aversive details of the traumatic event (American Psychiatric Association, 2013).

Post-traumatic stress disorder, as diagnosis for adult onset trauma, is often applied to traumatized children as well. Nevertheless, a PTSD diagnosis fails to account for the complex symptomatology that emerges following early chronic interpersonal traumatization (such as psychological maltreatment, physical and sexual abuse, neglect, separation from caregivers, traumatic loss, and the witnessing of domestic violence). In the attempt to more clearly delineate childhood trauma impact, the diagnostic construct “complex trauma” has been proposed to describe the consequences of early children’s exposure to multiple and prolonged interpersonal traumatic events that occur primarily within the caregiving system (van der Kolk, 2005).

Early interpersonal traumatization exerts a deleterious impact on children’s abilities to recognize, express, and regulate emotional states. Several studies have found that maltreated children exhibit less accurate recognition of emotions in others than non-maltreated children, and have a selective attentional bias toward the detection of anger (Pine et al., 2005). Physically abused children display a boundary shift for perceptual categories of anger (Pollak and Kistler, 2002), require less visual information to detect angry facial expressions (Pollak and Sinha, 2002), and recognize cues related to aggression earlier (Pollak et al., 2000, 2009). These attentional biases have been explained as the effects of exposure to a physically abusive environment, where anger may be associated with the potential for physical threat or harm to the child (Pollak et al., 2000). Other studies have demonstrated that, relative to non-maltreated comparison groups, maltreated children show a faster and more accurate response to fearful (Masten et al., 2008) and sad facial expressions (Leist and Dadds, 2009), and are more likely to perceive neutral faces as angry or sad (Leist and Dadds, 2009). Compared to both physically abused and non-maltreated children, neglected children (who have received less support from adults in learning to decode emotional signs) appear to have more difficulties in discriminating emotional expressions (Pollak et al., 2000).

Studies utilizing event-related potentials (ERPs) have given support to these behavioral findings. These studies have confirmed the detrimental effect of early maltreatment influences on neural processes associated with facial emotion recognition (Curtis and Cicchetti, 2011).

Neuroimaging research has extensively measured regional cerebral blood flow (rCBF) in adults (Francati et al., 2007) and children (Hart and Rubia, 2012) with PTSD, as compared to that of healthy controls. Investigations by positron emission tomography (PET) and single photon emission computed tomography (SPECT) have identified a hyper-reactivity of the amygdalae with acquisition of fear responses, and an impairment of the medial prefrontal cortex (mPFC) in properly regulating fear extinction, that could account for increased PTSD symptoms with traumatic reminders (Bremner, 2007; Looi et al., 2010).

With respect to the therapeutic approach, several studies have provided evidence for the clinical efficacy of Eye Movement Desensitization and Reprocessing (EMDR) therapy (Shapiro, 1989) in the treatment of PTSD (Shapiro, 2012). EMDR practice is guided by the adaptive information processing model (AIP model), (Shapiro, 2001) according to which a high level of disturbance related to traumatic experiences causes the information processing system to fail to properly process and store experience into the functional memory networks. The goal of EMDR is to access these dysfunctionally stored experiences and to transform them into adaptive ones, by stimulating the natural neural processes of memory consolidation (Shapiro, 2012). EMDR standardized protocol is structured in eight phases and requires the subject to focus on traumatic memories (*target*), while simultaneously being exposed to alternating bilateral stimulation (i.e., eye movements, tactile taps, or auditory tones). An adapted protocol, with age appropriate modifications, is used for children (**Table 1**), (Verardo, 2010).

**Table 1:** Overview of Eye Movement Desensitization and Reprocessing (EMDR) treatment for children.

EMDR Phases	Description
Phase 1: *Client History*	It involves history taking, client evaluation, identification of traumatic memories to be targeted, and treatment planning.
Phase 2: *Preparation*	The client is prepared for treatment, by stabilizing and increasing access to positive affects.
Phase 3: *Assessment*	The client is guided in accessing the perceptual, cognitive, affective, and somatic components of a specific disturbing memory. The client is asked to identify a preferred self-referential positive cognition and rates how valid it feels using the Validity of Cognition (VOC) scale, where 1 is not true and 7 is completely true (Shapiro, 2001). The client is also asked to rate the level of emotional disturbance using the Subjective Units of Disturbance (SUD) scale, where 0 is no disturbance and 10 is worst possible disturbance.
Phase 4: *Desensitization*	The client focuses on the memory for about 15–20 s (instead of 30 s as recommended in adults) while simultaneously engaging in therapist-directed bilateral stimulation (in children, especially eye movements or tactile taps), with lengthier sets during abreactions. After each set, the client reports any elicited material, which is then processed during bilateral stimulation, until the SUD score substantially decreases to zero.
Phase 5: *Installation*	The client is asked to focus on the positive cognition while thinking of the memory and engaging in new sets of bilateral stimulation, until the VOC score is 7.
Phase 6: *Body Scan*	Any residual physical disturbance associated with the memories are processed until the client reports that the body is clear and free of any disturbance.
Phase 7: *Closure*	Client’s stability at the completion of an EMDR session and between sessions is ensured.
Phase 8: *Reevaluation*	It occurs at the beginning of subsequent sessions to check whether results were kept unchanged or needed further reprocessing. In addition to targeting past traumas, EMDR also targets current triggers and related future anxieties.


The clinical efficacy of EMDR for treatment of trauma in adults has been documented by approximately 20 randomized controlled trials. In these studies, EMDR was compared to psychopharmaceuticals (van der Kolk et al., 2007) and several forms of psychotherapy, including exposure-based therapies (Bisson and Andrew, 2007) and trauma-focused cognitive-behavioral therapy (TFCBT) (Nijdam et al., 2012). A meta-analytic overview has documented the efficacy of EMDR both in children and in adolescents, demonstrating the incremental effect of EMDR when it was used along with cognitive-behavioral therapy (CBT; Rodenburg et al., 2009). On the basis of such empirical evidence, EMDR has been recommended as a first-line trauma treatment in the international practice guidelines of several organizations, including the American Psychiatric Association (2004).

Attempts to explain the mechanisms of action involved in EMDR have documented that eye movements may enhance memory retrieval and attentional flexibility, reduce the vividness, emotionality, and completeness of unpleasant or traumatic memories, decrease psychophysiological arousal and increase parasympathetic activity in people with PTSD symptoms (see Shapiro, 2014).

Recent evidence of the effectiveness of eye movements is provided by the working memory theories of EMDR (Gunter and Bodner, 2008; Maxfield et al., 2008; van den Hout and Engelhard, 2012). Working memory research has found that performance is impaired when participants engage in two simultaneous tasks that compete for the same limited working memory resources (Baddeley, 2000). In line with this, several studies have found that eye movements reduce the ability to hold a visual image in conscious awareness, resulting in the degradation of vividness (for a systematic review, see Lee and Cuijpers, 2013).

Another model to account for the possible role of eye movements that has received some empirical support is the orienting response theory. Consistent with such theory, eye movements activate an “investigatory reflex” in which, at first, a state of heightened alertness occurs; then, a reflexive pause produces de-arousal, allowing cognitive processes to become more flexible and efficient (Kuiken et al., 2001; Lee and Cuijpers, 2013).

Over the last few years, neuroimaging studies have been used to investigate the neurobiological substrate of EMDR in clinical practice (Pagani et al., 2013; Pagani and Cavallo, 2014). SPECT studies have documented significant changes in CBF patterns after EMDR (Lansing et al., 2005; Pagani et al., 2007), reflecting the recovered inhibitory role of the prefrontal cortex (PFC) in reducing amygdala hyperactivation in response to pathological stimuli that recall the traumatic event. Structural Magnetic Resonance Imaging (MRI) investigations have also provided some evidence that EMDR in PTSD may be associated with changes in limbic and paralimbic gray matter density, and with improvement of symptoms (Nardo et al., 2010; Bossini et al., 2012).

Several electroencephalography (EEG) investigations have explored the effects of bilateral stimulation of EMDR on brain activation/deactivation, evaluating patients before and after treatment. These studies have suggested that bilateral stimulation might be associated with: a reduced attention to novel stimuli and a diminished arousal level after therapy (Lamprecht et al., 2004); a depotentiation of fear memory synapses in the amygdala (Harper et al., 2009); and a decrease of the interhemispheric EEG coherence, which may foster the consolidation of traumatic memories, thereby reducing traumatic memory intrusions (Propper and Christman, 2008). Recently, the possibility to monitor by EEG, in real time, the relative neurobiological modifications occurring upon EMDR has been proposed (Pagani et al., 2011, 2012). The comparison between the EEGs of patients during the first and last session has showed, during the latter, a significant deactivation of the orbitofrontal and subcortical limbic structures, as well as a greater activation in the left temporo-occipital cortex (Pagani et al., 2012), suggesting that traumatic events had been processed at cognitive level following therapy.

While EMDR has been proven efficacious in restoring affective regulatory strategies in PTSD patients, much less is known about the effectiveness of EMDR in improving emotion processing in children with complex trauma. In keeping with this, the aim of the present preliminary study was to evaluate the effects of EMDR on neural processes associated with facial emotion processing, in a sample of children with histories of early and prolonged maltreatment perpetrated by their caregivers. We used high-density electroencephalography (hdEEG) before the start and after the conclusion of EMDR therapy, to explore the possible variations in children’s neural response to adults’ facial emotions. We predicted that EMDR would decrease the level of traumatic distress and empower the quality of emotional-adaptive functioning in children. Moreover, we hypothesized that, before treatment, exposure to adults’ facial emotions would induce maximal neural activations in the prefrontal and fronto-limbic regions, suggesting a dysfunctional ability in these children in the decoding of affective cues and regulating inner responses. We further hypothesized that, after EMDR treatment, exposure to adults’ facial emotions would be associated with higher neural firing in cognitively relevant regions, as a result of the processing of traumatic memories and relief from negative emotional experiences.

## Materials and Methods

### Participants

Ten Italian maltreated children (six boys and four girls) from low socioeconomic status backgrounds (Hollingshead, 1957) took part in the study. Seven children were living with their mothers, one child was living with the father, one with the maternal aunt, and one with both parents.

The children were aged between 7 and 12 and were recruited from the Centro Provinciale Giorgio Fregosi – Spazio Sicuro of Rome, where they were sent by social services or directly by the Court for clinical evaluation. All children were clinically diagnosed as suffering from complex trauma, reporting at least two of the early and prolonged forms of maltreatment listed in **Table 2.**

**Table 2:** Demographic and maltreatment characteristics.

**Mean child age (years)**	9,53
	(±1,62; range 7–12)
**Sex of the child**	
Female	4
Male	6
**Mean custodial caregivers’ age**	44,97
	(±10,09; range 35–72)
**Custodial caregivers’ marital status**	
Married	1
Separated, divorced, or widowed	9
**Hollingshead’s two factor index of social position**	58,10
(Hollingshead, 1957)	(±17,84; range 29–80)
**Maltreatment experiences**	
Psychological maltreatment, witnessing domestic violence, and neglect	2
Sexual abuse, neglect, and psychological maltreatment	1
Physical abuse, sexual abuse, psychological maltreatment, and neglect	1
Sexual abuse and psychological maltreatment	1
Psychological maltreatment and witnessing domestic violence	1
Physical abuse and witnessing domestic violence	1
Psychological maltreatment and neglect	1
Physical abuse, sexual abuse, and psychological maltreatment	1
Sexual abuse, physical abuse, psychological maltreatment, and neglect	1


Children were examined before (T0) and within 1 month after the conclusion of EMDR therapy (T1), by means of psychological assessment and hdEEG recordings. At the time of the hdEEG recordings, children were medically and neurologically healthy, and free of all substances and medications.

The study has been carried out in accordance with the recommendations of the Ethics Committee of the Institute of Cognitive Sciences and Technologies of the Italian National Research Council (CNR) of Rome. Prior to data collection, custodial caregivers of all children received complete information concerning the rationale and effectiveness of EMDR and the study procedures, and gave written informed consent for children’s participation, in accordance with the Declaration of Helsinki.

### EMDR Procedure

Therapy was administered once a week for 8–10 weeks at the Centro Provinciale Giorgio Fregosi – Spazio Sicuro of Rome, by a therapist who is a member of the Italian EMDR Association. Before EMDR, children received a simple and clear explanation of what would happen during therapy (that is, focusing on the memory of a particularly negative event, while performing left–right–left eye movements or during tactile tapping administered by the therapist). The therapist used a symptom-focused approach to identify the traumatic experiences that were directly linked to the children’s current triggers and symptoms. This strategy was extremely useful in identifying those memories, embedded within the multifaceted context of complex trauma, that were mostly activated and relevant with regard to the children’s current dysfunctions. At the beginning of the EMDR sessions, children were asked to focus on these primary traumatic memories, while simultaneously following the therapist-directed bilateral stimulation, using eye movements or, to a lesser extent, tactile taps (the latter were used only when children were not able to keep their eyes open when recalling traumatic events). Once the most activated traumatic memories were desensitized, the EMDR protocol was applied to the other relational traumas experienced by the children, as well as to current triggers and related future anxieties.

### Clinical Scales

#### Traumatic Stress and Related Psychological Symptomatology

Children completed the alternate version of the Trauma Symptom Checklist for Children (TSCC-A; Briere, 1996; Italian validation by Di Blasio et al., 2011), a questionnaire used for the detection of a cluster of psychological consequences that might have been triggered by traumatic events. TSCC-A consists of two validity scales (Under response and Hyper response) and five clinical scales (Anxiety, Depression, Anger, Post-traumatic Stress, and Dissociation).

#### Children’s Behavioral/Emotional Problems and Competencies

Custodial caregivers completed the Child Behavior Checklist/4-18 (CBCL/4-18; Achenbach, 1991; Italian validation by Frigerio et al., 2004), a questionnaire which provides a report of children’s and adolescents’ competencies and behavioral problems. The CBCL/4-18 yields scores for eight subscales (Social Withdrawal, Somatic Complaints, Anxiety/Depression, Social Problems, Thought Problems, Attention Problems, Rule-Breaking Behavior, and Aggressive Behavior). The sum is the Total Problem scale. The CBCL/4-18 also allows the examination of two broad groupings of syndromes: Internalizing Problems and Externalizing Problems.

The validation studies of the Italian versions of the questionnaires have shown adequate psychometric properties for both the TSCC-A (see Di Blasio et al., 2011) and the CBCL/4-18 (see Frigerio et al., 2004).

### hdEEG Stimuli and Procedure

**FIGURE 1:**
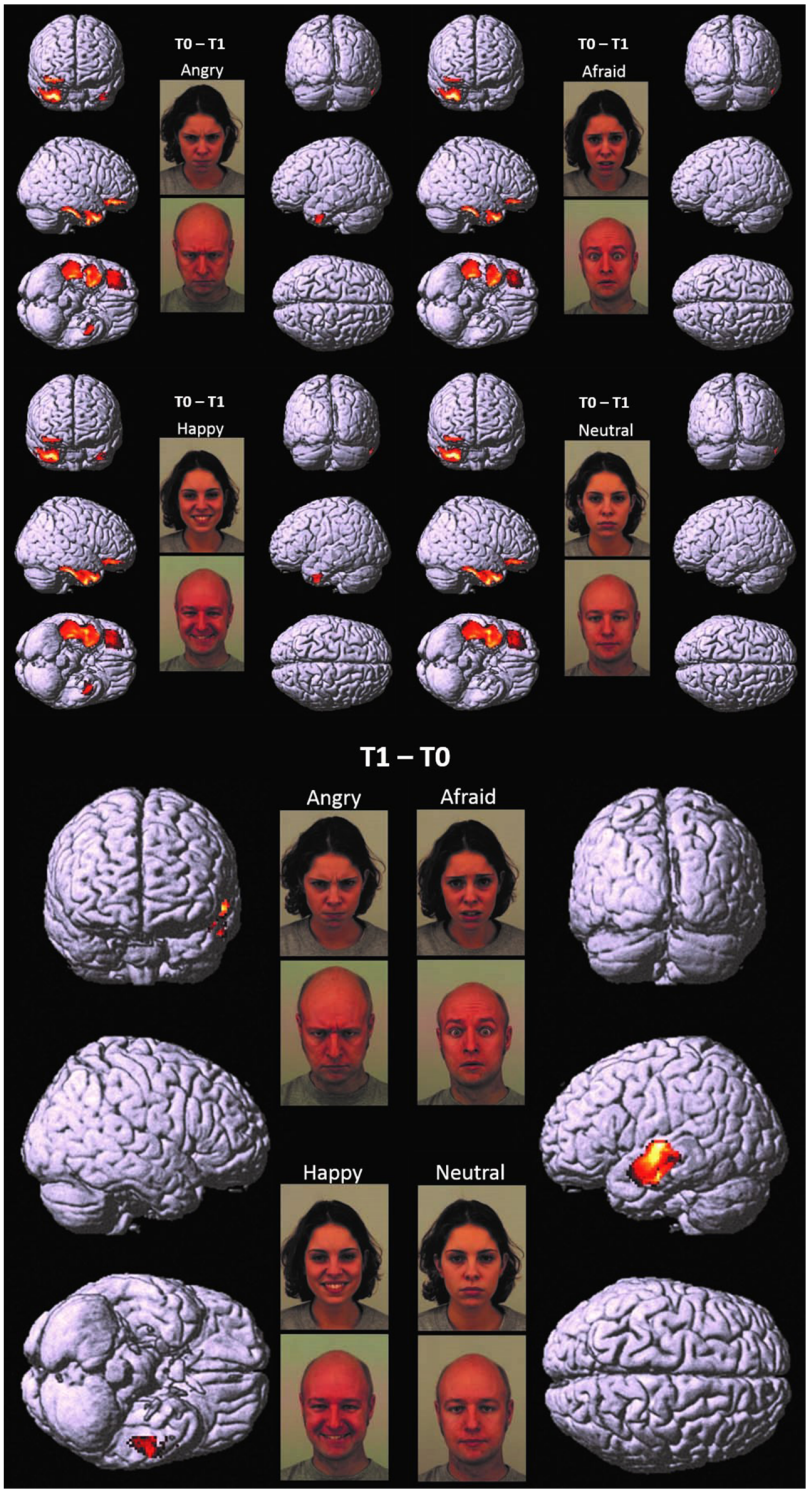
**T0 – T1 and T1 – T0.** Cortical representation of the cluster of voxels in which the hdEEG signal was higher at T0 as compared to T1 **(Top)** and at T1 as compared to T0 **(Bottom)**. For T0 – T1, we reported the patterns of activations in response to angry, afraid, happy, and neutral emotions, respectively. For T1 – T0, we reported only one representative pattern of activation (referred specifically to angry faces), since brain responses were elicited in a very similar fashion for all the presented emotions. Regional details are presented in **Table 4.**

At each hdEEG recording, angry, afraid, happy, and neutral faces (40 of each) were presented *full-screen* on a 15″ color monitor, in randomized and unpredictable order. The pictures represented colored frontal head shots of 40 adult amateur actors (50% men and 50% women), aged between 20 and 30, taken from the Karolinska Directed Emotional Faces Series (KDEF; Lundqvist et al., 1998), (**Figure 1**). The pictures were uniform with regard to brightness, shading, and size of the head. Each picture was presented once for 1500 ms, with an inter-trial interval (ITI) of 1000 ms. Children were instructed to simply look at the pictures and pay attention to adults’ emotions.

### hdEEG Recording

Stimuli presentation was controlled through a PC running E-Prime Software, Version 2.0 (E–Prime^®^2.0, 2012). Two hundred and fifty six channel dense array EEG was recorded using the Geodesic Sensor Net (Electrical Geodesics Inc., Eugene, OR, USA), owned by the Department of Dynamic and Clinical Psychology of “Sapienza” University of Rome. The electrodes net montage required approximately 15 min and was well tolerated by the children. Data were digitized at 512 Hz. All channels were referenced to the vertex electrode (Cz). Impedance was kept below 10 kΩ.

### Data Analysis

#### Psychological Analysis

Paired *t-*tests were performed to compare the scores of TSCC-A and CBCL/4-18 before and after EMDR treatment in children.

#### hdEEG Processing and Analysis

High-density electroencephalography raw files were exported in binary format and converted by the Statistical Parametric Mapping 8 (SPM8) into “mat files”. The latter were co-registered to the virtual template corresponding to the hdEEG headset. Based on published literature, epoching was performed for each trial on the temporal window 100–400 milliseconds and the resulting files high-pass filtered at 0.1 Hz. Artifacts were removed by a Flat-segment method and resulting files underwent Robust Averaging. The analyzed range of the frequency bands was 0–30 Hz.

Source analysis was performed by space modeling, data co-registration, forward computation, inverse reconstruction, and creation of a three-dimensional Neuroimaging Informatics Technology Initiative (3D NIfTI) image, according to Litvak et al. (2011).

For each subject NIfTI images were generated by 40 trials for each experimental condition (angry, afraid, happy, and neutral).

The flexible factorial routine of SPM8 was used to compare within-subjects hdEEG signals at T0 and T1. Whole brain cortical activity was then covaried to TSCC-A and CBCL/4-18 scores, adding the covariate scores to the flexible factorial design of SPM8 one at a time. Positive and negative correlations were assessed.

The statistical threshold was set at *p* < 0,05 corrected for multiple comparisons with the Family Wise Error (FWE_corr_) option at cluster and voxel level, only accepting cluster sizes exceeding 125 (5×5×5) voxels.

Anatomical regions were identified by Talairach Client 2.4.3, after converting the output isocenters coordinates to Talairach space by using the subroutine implemented by Matthew Brett (http://brainmap.org/index.html).

## Results

### Clinical Scales

Significant differences were found for TSCC-A at T1 as compared to T0, highlighting a decrease of scores on Depression and Post-traumatic Stress clinical scales. Scores on CBCL/4-18 also decreased significantly in children at T1. Significant differences across time were found in four syndrome scales, as well as in Internalizing, Externalizing, and Total Problem scales (**Table 3**).

**Table 3:** Pre vs. post EMDR treatment: mean (SD) and statistically significant differences in CBCL/4-18 and TSCC-A scores in children.

Psychological measures		T0 *M (SD)*	T1 *M (SD)*	*T*	*p*
TSCC-A	Post-traumatic stress	10,10 (5,36)	6,20 (3,16)	2,345	0,044
	Depression	5,70 (3,50)	3,40 (2,27)	2,815	0,020
CBCL/4-18	Somatic complaints	4,90 (3,14)	2,70 (1,89)	2,659	0,026
	Anxiety/Depression	8,80 (6,56)	6,80 (5,05)	2,268	0,050
	Thought problems	6,60 (6,98)	4,00 (4,37)	2,286	0,048
	Aggressive behavior	12,30 (10,30)	8,30 (8,30)	4,671	0,001
	Internalizing problems	16,80 (11,75)	12 (7,85)	3,191	0,011
	Externalizing problems	16,40 (14,88)	12,20 (12,25)	3,215	0,011
	Total problem	56,70 (46,39)	41,90 (36,06)	2,956	0,018


### hdEEG

#### Children at T0 vs. Children at T1

At T0 as compared to T1, a significantly higher cortical activation was found during the observation of angry, afraid, happy, and neutral expressions in inferior/medial PFC: specifically, in the inferior frontal gyrus (IFG, BA 47) and in the orbitofrontal cortex (OFC, BA 11). Significantly higher activations were also found at T0 as compared to T1 in the temporal pole (TP, BA 38) and in the inferior temporal gyrus (ITG, BA 20). These prefrontal and temporal activations were mainly found in the right hemisphere, with the exception of ITG, which was activated bilaterally both by angry and happy emotions.

#### Children at T1 vs. Children at T0

At T1 as compared to T0, significantly higher left-sided activations were found in middle and superior temporal gyri (MTG, BA 21; STG, BA 22) in response to all emotions (**Table 4**; **Figure 1**).

**Table 4:** Pre vs. post EMDR treatment: regions in which significant differences were found during the observations of the emotional expressions.

Emotions	Region	T0 – T1					T1 – T0				
			
		*x*	*y*	*z*	*T*	Cluster size	*x*	*y*	*z*	*T*	Cluster size
Angry	rIFG	32	34	–10	7,07	478	–	–	–	–	–
	rOFC	34	36	–14	7,12	478	–	–	–	–	–
	lITG	–40	2	–42	5,79	182	–	–	–	–	–
	rITG	42	–26	–20	9,70	1664	–	–	–	–	–
	rTP	38	14	–40	8,44	1664	–	–	–	–	–
	lMTG	–	–	–	–	–	–58	–6	–6	15,12	968
	lSTG	–	–	–	–	–	–58	–10	0	15,14	968
Afraid	rIFG	32	34	–10	5,70	300	–	–	–	–	–
	rOFC	34	36	–14	5,73	300	–	–	–	–	–
	lITG	–	–	–	–	–	–	–	–	–	–
	rITG	42	–26	–20	9,53	753	–	–	–	–	–
	rTP	38	14	–40	8,18	724	–	–	–	–	–
	lMTG	–	–	–	–	–	–58	–6	–6	14,66	948
	lSTG	–	–	–	–	–	–58	–10	0	14,68	948
Happy	rIFG	32	34	–10	6,99	446	–	–	–	–	–
	rOFC	34	36	–14	7,03	446	–	–	–	–	–
	lITG	–38	2	–40	5,92	203	–	–	–	–	–
	rITG	42	–24	–20	10,85	2030	–	–	–	–	–
	rTP	40	12	–42	9,08	2030	–	–	–	–	–
	lMTG	–	–	–	–	–	–58	–6	–6	13,85	920
	lSTG	–	–	–	–	–	–58	–10	0	13,87	920
Neutral	rIFG	32	34	–10	6,61	418	–	–	–	–	–
	rOFC	34	46	–14	6,65	418	–	–	–	–	–
	lITG	–	–	–	–	–	–	–	–	–	–
	rITG	42	–24	–20	9,77	1908	–	–	–	–	–
	rTP	38	12	–40	9,08	1908	–	–	–	–	–
	lMTG	–	–	–	–	–	–58	–6	–6	14,31	937
	lSTG	–	–	–	–	–	–58	–10	0	14,33	937


*Only peaks of clusters corrected for multiple comparison at cluster level *p* < 0,05 are reported. IFG, inferior frontal gyrus; OFC, orbitofrontal cortex; ITG, inferior temporal gyrus; TP, temporal pole; MTG, middle temporal gyrus; STG, superior temporal gyrus; r, right; l, left.*

#### Correlation Analyses

Positive correlations were found between: (i) TSCC-A Post-traumatic Stress scores and brain activation in lTP and bilateral precuneus; (ii) TSCC-A Depression scores and brain activation in bilateral OFC, rIFG, and rITG; (iii) CBCL/4-18 Anxiety/Depression scores and brain activation in lIFG, bilateral middle frontal gyrus (MFG, BA, 10), and bilateral OFC. TSCC-A Post-traumatic Stress and Depression scores negatively correlated with brain activation in both lMTG and lSTG (**Table 5**).

**Table 5:** Brain regions whose activity positively correlated with TSCC-A and CBCL/4-18 scores.

Psychological measures	Region	*x*	*y*	*z*	*T*	Cluster size
TSCC-A Post-traumatic Stress	lTP	–34	14	–34	7,89	751
	r precuneus	10	–60	54	6,97	207
	l precuneus	–8	–62	50	6,08	165
TSCC-A Depression	rOFC	28	54	–14	11,39	1252
	lOFC	–30	54	–14	7,75	736
	rIFG	40	22	–16	9,66	1252
	rITG	40	–22	–26	10,01	1803
CBCL-4/18 Anxiety/Depression	lIFG	–46	28	–14	6,61	271
	rMFG	38	52	0	6,19	613
	lMFG	–38	34	–16	6,91	271
	rOFC	42	48	–10	7,47	613
	lOFC	–35	40	–14	6,87	271


*Only peaks of clusters corrected for multiple comparison at cluster level *p* < 0,05 are reported. TP, temporal pole; OFC, orbitofrontal cortex; IFG, inferior frontal gyrus; ITG, inferior temporal gyrus; MFG, middle frontal gyrus. r, right; l, left.*

Lastly, significant negative correlations were also found between the CBCL/4-18 Anxiety/Depression scores and brain activation in rITG (**Table 6**).

**Table 6:** Brain regions whose activity negatively correlated with TSCC-A and CBCL/4-18 scores.

Psychological measures	Region	*x*	*y*	*z*	*T*	Cluster size
TSCC-A Post-traumatic Stress	lMTG	–58	–6	–6	8,70	694
	lSTG	–58	–10	0	8,71	694
TSCC-A Depression	lMTG	–58	–6	–6	6,65	461
	lSTG	–58	–10	0	6,68	461
CBCL-4/18 Anxiety/Depression	rITG	48	–8	–40	3,71	301


*Only peaks of clusters corrected for multiple comparison at cluster level *p* < 0,05 are reported. MTG, middle temporal gyrus; STG, superior temporal gyrus; ITG, inferior temporal gyrus. r, right; l, left.*

## Discussion

Early maltreatment alters the trajectories of brain development, decreasing the functionality of cerebral regions related to emotion processing (Curtis and Cicchetti, 2011; Hart and Rubia, 2012). In the attempt to explore these aspects, we used hdEEG to assess the impact of EMDR on traumatized children, taking into account neural responses to adults’ facial emotions, as well as the levels of traumatic distress and emotional-adaptive functioning.

Before EMDR (T0 vs. T1), significant cortical activations were found in inferior/medial PFC as well as TP and ITG, with a prevalent lateralization in the right hemisphere. After EMDR therapy (T1 vs. T0), we found a reduced activity in these cerebral regions and a significant increase of cortical activation in lMTG and lSTG.

The significantly higher activation found in children at T0 as compared to T1 in the right regions of inferior/medial PFC – OFC and IFG, respectively, – may reflect the dysfunctional ability in these children in decoding affective cues and regulating inner responses to adults’ emotions (**Table 4**; **Figure 1**). These findings are coherent with those of previous studies, which indicate the impairment of mPFC in regulating the response of the limbic system (including the amygdala and the related nuclei and circuitry) to stimuli that resemble traumatic events (Bremner, 2007; Looi et al., 2010; Pagani et al., 2011, 2012). It has been suggested that early and prolonged stress may result in more prefrontal cortical catecholamine concentration (especially norepinephrine and dopamine) than is functionally necessary to cope with the stressors. Extreme levels of dopamine, in particular, may impair frontal inhibition of the limbic system, exaggerating attention and vigilance toward cues that are experimented as potentially traumatic (De Bellis et al., 2011). Prefrontal activation is implicated in the introspective evaluation of self-generated material (Ramnani and Owen, 2004) and in the decoding of the emotional value of incoming information (Steele and Lawrie, 2004). Moreover, PFC is involved in autobiographical memory retrieval (Tulving et al., 1994) and in the suppression of unwanted memories during autobiographical recall (Anderson et al., 2004). All these functions seem to be exaggerated in patients before EMDR therapy, since the self-referential emotional contents cause larger activation in rPFC than in normal individuals or in patients after having processed the traumatic event (Pagani et al., 2012).

At T0 as compared to T1, higher response to all adult emotions was also found in rTP, a paralimbic area which, in conjunction with inferior frontal lobe structures, is activated during autobiographical memory retrieval (Engdahl et al., 2010). Previous studies indicate that activation in rTP increases when subjects attend more “socially relevant” dimensions of a visual display, such as emotions, therefore modulating the amygdala response to threat/fearful stimuli (Olson et al., 2013). Moreover, rTP (together with rmPFC) is activated when inferring the thoughts and the feelings of other people is used to guide personal social behaviors (Olson et al., 2013).

Lastly, significantly higher activity was found at T0 as compared to T1 in ITG. This region also plays a key role in memory recall; moreover, in conjunction with the adjacent fusiform gyrus, it is implicated in face perception and recognition, receiving greater contribution from the amygdala especially during the processing of fearful expressions (Schupp et al., 2004).

Before EMDR, cerebral responses in children did not vary according to the specific valence of the presented emotions. These results are coherent with scientific literature on complex trauma, according to which children who are exposed to early and prolonged traumatic events often experience intense negative affects (such as rage, betrayal, fear, resignation, defeat, and shame), associated with a persistent sensibility to negative emotions. The aim is to prevent potentially traumatic experiences. Such enhanced sensitivity causes a long-term emotional dysregulation, characterized by over- or/and under reactivity to emotional minor stimuli that would have no significant impact on non-maltreated children (van der Kolk, 2007). In line with this, research has shown that maltreated children have poor discriminatory abilities for different facial emotions and misinterpret all emotional faces (including neutral and happy ones) as being threatening or as a mask for more malevolent emotions (Pollak et al., 2000; Leist and Dadds, 2009; van Harmelen et al., 2013).

Further relevant neurobiological results which emerged in our study were the differences shown between the cortical activation at T1 as compared to T0 (**Table 4**; **Figure 1**). In this comparison, we found a reduced activity after EMDR intervention in the right regions of inferior/medial PFC, as well as rTP and ITG, and a significant increase of cortical activation at T1 in lMTG and lSTG.

These temporal areas play a key role in social cognition, since they encode and retrieve autobiographical memory, process concepts with social–emotional content, and associate highly perceptual and emotional information to form a personal semantic store (Olson et al., 2013). The MTG modulates emotional processes, such as sensitivity to threatening cues, anxiety and mood disorders (Davidson, 2004). It has been suggested that early and prolonged traumatic experiences may impair the functioning of this region, encompassing the amygdala and the hippocampus (Maheu et al., 2010).

Even though from different perspectives, current theories on the mechanisms of action involved in EMDR provide possible explanations of the neurobiological changes we observed over time in children.

According to the AIP model (Shapiro, 2001), EMDR allows to access traumatic memories which are dysfunctionally stored, transforming them into adaptive ones, by stimulating the natural neural processes of memory consolidation (Shapiro, 2012). Once the memory retention of the traumatic event can move from an implicit subcortical status to an explicit cortical one, the traumatic memories and their related emotions may be elaborated at higher cognitive level. Coherently with the results of previous researches (Pagani et al., 2012), our study seems to indicate that, after EMDR, children use high-order cognitive resources while processing emotion expressions.

The working memory theories of EMDR may provide a further significant contribution to the explanation of the results of our study. Working memory allows the individuals to access memories, retrieve related material, compare this to what they are currently perceiving, integrate new material with old material, and form new understandings to guide future behaviors (Baddeley, 2000). Research in EMDR domain has consistently found that the vividness and emotionality of memory is reduced when individuals are simultaneously engaged in performing eye movements and focusing on a traumatic image, since both tasks make demands on the same limited working memory resources (Gunter and Bodner, 2008; Maxfield et al., 2008; van den Hout and Engelhard, 2012). As a result, the target memory is perceived as less distressing and is more likely to be processed from an observational or detached perspective (Maxfield et al., 2008). In our study, such “distancing” effect (Lee et al., 2006) may have allowed children to regulate inner responses to adults’ emotions through the use of higher cognitive processes.

It is important to notice that the increase in efficiency of cognitive processes is a key point of the orienting response theory of EMDR (Armstrong and Vaughan, 1996; Kuiken et al., 2001; Lee and Cuijpers, 2013). Consistent with such theory, eye movements may induce attentional flexibility, thereby facilitating the re-elaboration of the subjective representation of traumatic experiences.

As at T0, activations at T1 in cognitively relevant areas did not vary according to the specific valence of the emotional stimuli. This result shows that EMDR did not affect the children’s capacity to discriminate different emotional expressions: it rather appears that EMDR may have contributed in globally restoring the processing of emotional cues (regardless of their type), resulting in all faces being processed and contextualized in semantic memory in the same way.

Interestingly, the comparisons between T1 and T0 in children showed different outcomes with a clear lateralization toward the left hemisphere after EMDR. According to the emotional asymmetry theory, the right hemisphere is dominant over the left for emotional expression and perception, while the left hemisphere has an important role in explicating emotions at a semantic level (Alves et al., 2008). The prominent activation found at T1 in left temporal areas might then be the result of a high-order cognitive processing of traumatic memories, reaching the explicit state after EMDR, along with a significant restraint of negative emotional experiences. These findings are noticeably similar to those shown in a previous study, in which EEG was used to monitor neuronal activation in adults throughout EMDR sessions (Pagani et al., 2012).

Complex trauma poses children at risk for many psychopathological outcomes, among which internalizing and externalizing problems have been extensively studied, in association with the severity and persistence of trauma-related symptoms. TSCC-A and CBCL/4-18 are commonly administered in pre- and post-treatment, as measures of treatment outcome. This approach is particularly relevant in studies on effectiveness of psychotherapy for traumatized children (Farkas et al., 2010).

In this study, the decrease in both CBCL/4-18 and TSCC-A scores indicated the improvement of children’s behavioral and emotional problems, along with the reduction of post-traumatic distress. Among these changes, the striking decrease in depressive symptomatology (as measured by TSCC-A Depression and CBCL/4-18 Anxiety/Depression scales) is coherent with the description of “trauma spectrum psychiatric disorders” (Bremner, 2002), that includes mild to severe depression and anxiety disorder. These findings suggest the importance to take into account these psychopathological symptoms as a further indication of EMDR treatment in childhood trauma spectrum.

Correlation analyses were performed in order to detect brain regions whose activity was linked to children’s traumatic symptom-related and emotional-adaptive problem scores. Significant correlations were found between TSCC-A Post-traumatic Stress, TSCC-A Depression, and CBCL/4-18 Anxiety/Depression scores and the same regions whose activity significantly differed between pre- and post-treatment. We found significant positive correlations between these clinical scales and brain activation in the right fronto-temporal limbic regions which are known to be involved in affective dysregulation in response to stimuli that resemble traumatic experiences (Tulving et al., 1994; Anderson et al., 2004; Bremner, 2007; Engdahl et al., 2010; Looi et al., 2010; Pagani et al., 2011, 2012), (**Table 5**). Moreover, negative correlations were also found between the same scores and brain activation in left medial/superior temporal areas implicated in high-order cognitive processing (Pagani et al., 2012; Olson et al., 2013), (**Table 6**). Such correlations confirm the role of the above-mentioned clinical scales in the assessment of childhood trauma, and highlight the neurobiological correlates of EMDR therapy.

## Conclusion

To our knowledge, this is the first study that has investigated the effects of EMDR on brain responses to adults’ emotions on children with complex trauma. Our preliminary findings have demonstrated that after EMDR, early maltreated children show increased activity in areas implicated in high-order cognitive processing when passively viewing pictures of emotional expressions. These changes are associated with the decrease of depressive and traumatic symptoms, and with the improvement of emotional-adaptive functioning over time. These results may have relevant implications in clinical practice, suggesting the importance of focusing interventions with traumatized children on cognitive processing of emotions.

The major constraints of this study are the relatively small number of the investigated subjects and the absence of a control group (e.g., children treated with other forms of psychotherapy). Nevertheless, low sample size lied in the difficulty in applying hdEEG to traumatized children and in the high costs and complicated methodologies of neuroimaging investigation. Another limitation relates to the fact that we used hdEEG measures only, not combining them with behavioral (e.g., reaction times) or self-report measures of emotion processing (e.g., subjective evaluation of stimulus salience). Correlations between hdEEG data and behavioral and/or self-report measures of emotion processing would have provided a more articulated picture of changes in brain activity after EMDR.

In the future, recruitment of patients treated with different psychotherapeutic approaches may increase the robustness of the results, adding a between-subjects analysis to the comparison of patients at T0 and T1. Moreover, the inclusion of behavioral and/or self-report measures of emotion processing may further contribute to clarifying the specific effects of EMDR on cerebral areas related to emotion processing in children with complex trauma.

## Author Contributions

CT: conceived the work and designed the hdEEG study, acquired hdEEG data, analyzed psychological data, and played a leading role in the composing of this paper. As first author, she is primarily accountable for all aspects of the work. MP: monitored data acquisition, supervised hdEEG data analyses, provided a substantial contribution to the interpretation of data, and wrote parts of the manuscript (in particular, “hdEEG Processing and Analysis” and parts of the “Discussion”). He revised the paper for intellectual content and approved its final version to be published. He agreed to be accountable for all aspects of the work and to ensure that questions related to the accuracy or integrity of any part of the work were appropriately investigated and resolved. PF: served as the expert in SPM analyses, provided a substantial contribution to the interpretation of hdEEG data, and drafted parts of the paper (most notably, **Tables 4** and **5** in “Results”). He approved the final version of the paper to be published, and agreed to be accountable for all aspects of the work and to ensure that questions related to the accuracy or integrity of any part of the work were appropriately investigated and resolved. AS: monitored the recruitment of subjects and provided a substantial contribution to the interpretation of data. She revised the paper for intellectual content and approved its final version to be published. She agreed to be accountable for all aspects of the work and to ensure that questions related to the accuracy or integrity of any part of the work were appropriately investigated and resolved. GN: contributed substantially to the conception of the work and provided a substantial contribution help to the interpretation of data. He revised the paper for intellectual content and approved its final version to be published. He agreed to be accountable for all aspects of the work and to ensure that questions related to the accuracy or integrity of any part of the work were appropriately investigated and resolved. AS: contributed substantially to the recruitment of subjects, administered EMDR therapy, acquired psychological data, and approved the final version of the paper to be published. She agreed to be accountable for all aspects of the work and to ensure that questions related to the accuracy or integrity of any part of the work were appropriately investigated and resolved. LI: provided substantial contributions to the design of the hdEEG study, as well as to the acquisition and interpretation of data. He approved the final version of the paper to be published, and agreed to be accountable for all aspects of the work and to ensure that questions related to the accuracy or integrity of any part of the work were appropriately investigated and resolved. AV: contributed substantially to the recruitment of subjects and provided a substantial contribution to the interpretation of data. She revised the paper for intellectual content and approved its final version to be published. She agreed to be accountable for all aspects of the work and to ensure that questions related to the accuracy or integrity of any part of the work were appropriately investigated and resolved. IF: provided a substantial contribution to the interpretation of data and counseled in essential questions about EMDR therapy. She revised the paper for intellectual content and approved its final version to be published. She agreed to be accountable for all aspects of the work and to ensure that questions related to the accuracy or integrity of any part of the work were appropriately investigated and resolved. MA: contributed substantially to the conception and design of the work. He drafted the work, revised it for intellectual content and approved its final version to be published. He agreed to be accountable for all aspects of the work and to ensure that questions related to the accuracy or integrity of any part of the work were appropriately investigated and resolved.

## Conflict of Interest Statement

The authors declare that the research was conducted in the absence of any commercial or financial relationships that could be construed as a potential conflict of interest.
